# Supplementary material for: Adding salt to expand voltage window of humid ionic liquids
Source: Nat Commun. 2020 Nov 16;11:5809. doi: 10.1038/s41467-020-19469-3 (PMC7670447; doi:10.1038/s41467-020-19469-3)
Supplement: Supplementary file 1 — Supplementary Information [file 41467_2020_19469_MOESM1_ESM.pdf]

Supplementary Information

Adding salt to expand voltage window of humid ionic liquids

Chen *et al.*

Table of Contents

Supplementary Note 1: Simulation setup 3

Supplementary Note 2: Preparation of electrolytes 5

Supplementary Note 3: HOPG surface characterization 8

Supplementary Note 4: Interfacial structure of electrolyte/electrode interfaces 9

Supplementary Note 5: Definition of bound water 14

Supplementary Note 6: Association of IL and water with interfacial Li^+ 15

Supplementary Note 7: OCP and scan rate effect 17

Supplementary Note 8: Effect of the molar salt-water ratio 20

Supplementary Note 9: IR spectrum and H-bond of water 21

Supplementary Note 10: Umbrella sampling method 28

Supplementary Note 11: Arrangement of interfacial water 30

Supplementary Note 12: Validation by more ionic liquids 35

Supplementary Note 1: Simulation setup

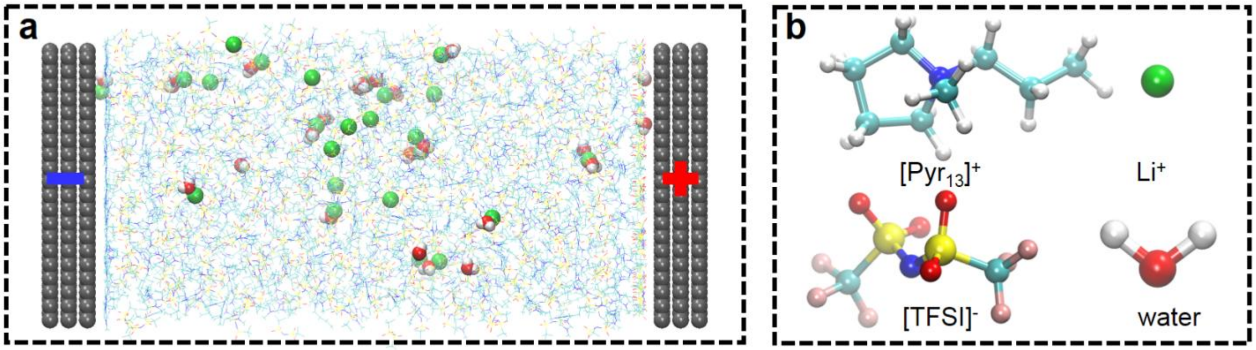

Supplementary Figure 1 | Schematic of molecular dynamics simulation. a, Snapshot of humid ionic liquids with adding salt between two planar carbon electrodes. **b,** Molecular structure of cation (1-methyl-1-propylpyrrolidinium, $[Pyr_{13}]^{+}$), anion (bis(trifluoromethylsulfonyl)imide, $[TFSI]^{-}$), water and lithium ion (Li^{+}). In molecular dynamics simulation system, z-direction is vertical to the electrode surface.

Supplementary Table 1. Setup parameters of molecular dynamics simulation systems. Number of cation, anion, water molecules and Li^+ ions, the molar salt-water ratio, and the distance between two electrodes (L), as well as the water content in each simulation system. Cation in the table means the cation in ionic liquids (ILs), i.e., 1-methyl-1-propylpyrrolidinium ($[Pyr_{13}]^+$) and 1-butyl-3-methylimidazolium ($[Bmim]^+$); anion in the table means bis(trifluoromethylsulfonyl)imide ($[TFSI]^-$) in this work.

		**MD system**	**cation**	**anion**	**water**	**Li^+**	**salt-water ratio**	**L (nm)**	**water content (ppm)**
$[Pyr_{13}]$ [TFSI]	Humid IL	system1	420	420	42	0	0	10.0	4392
	Salt-in-humid IL	system2	420	462	42	42	1:1	10.6	4105
		system3	420	504	42	84	2:1	11.1	3853
[Bmim] [TFSI]	Humid IL	system4	430	430	52	0	0	10.0	5168
	Salt-in-humid IL	system5	430	430	52	52	1:1	10.8	4775

Note: the water-IL ratio in humid IL is equal to that in salt-in-humid IL, and the smaller water concentration in salt-in-humid IL is because of the increase in total mass of electrolyte with adding salt.

Supplementary Note 2: Preparation of electrolytes

Before preparation of pure ILs, humid ILs and salt-in-humid ILs, it is necessary to explore hygroscopicity of ILs. To examine the capacity of ILs [Pyr_{13}][TFSI] and [Bmim][TFSI] absorbing water molecules from environment, experiments were carried out under almost constant humidity (43.72 ±2.45% humidity for [Pyr_{13}][TFSI]; 43.00 ±1.68% for [Bmim][TFSI]). ILs were purified through ultra-pure water and then vacuum-dried for 24 hours at 80 ℃ in a glovebox filled with ultrapure Argon (Linde Industrial Gases, 99.999%) to remove as much water as possible. Subsequently, ILs were exposed to room environment to examine the water content change. Water contents were determined by Karl Fisher Coulometer (Metrohm, KF-831).[1] **Supplementary Figure 2** shows the hygroscopicity of ILs [Pyr_{13}][TFSI] and [Bmim][TFSI]. After a 48-hour exposure to air, the water content of [Pyr_{13}][TFSI] reaches to ~4896 ppm, and the water content for [Bmim][TFSI] is up to ~5474 ppm.

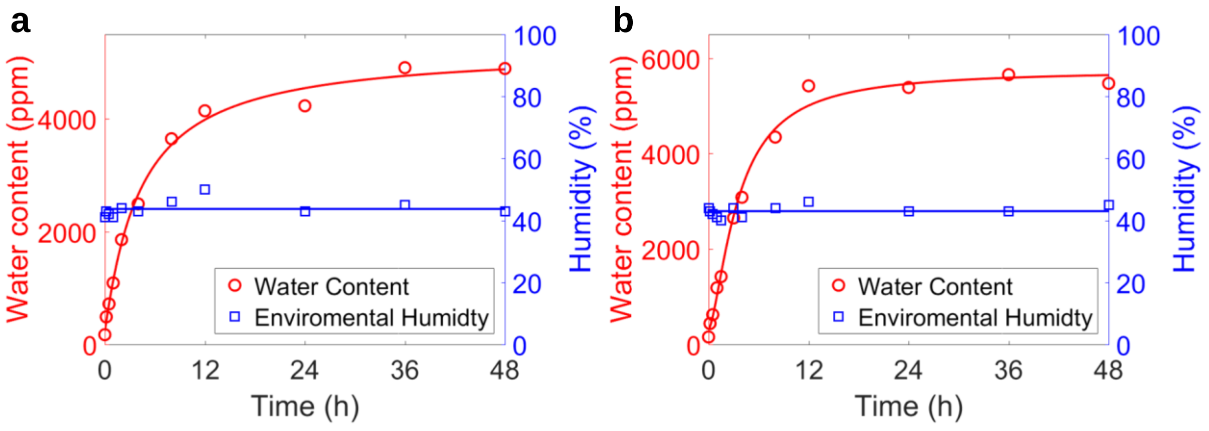

Supplementary Figure 2 | Hygroscopicity of ionic liquids in humid air. Water content in 1-methyl-1-propylpyrrolidinium bis(trifluoromethylsulfonyl)imide ([Pyr_{13}][TFSI]) **(a)** and 1-butyl-3-methylimidazolium TFSI ([Bmim][TFSI]) **(b)** as a function of time. Red lines are to guide the eyes; blue lines represent the averaged humidity during the measurements.

To prepare the humid IL, 6.4 μL (0.36 mmol) ultrapure water was added into dry [Pyr_{13}][TFSI] (1.00 mL, ρ = 1.43 g cm^{-3}, 3.50 mmol), and then the mixture was stirred till homogeneous solution was formed. The molar ratio of water to IL is around 1:9.7. Then the water content was ~4474 ppm, close to ~4392 ppm adopted in MD simulation. As for salt-in-humid IL, 6.4 μL (0.36 mmol) ultrapure water and 0.1033 g (0.36 mmol) vacuum dried LiTFSI (i.e., the molar salt-water ratio is 1:1) were added into dry [Pyr_{13}][TFSI] (1.00 mL, 1.43 g cm^{-3}, 3.50 mmol), and then the mixture was stirred till homogeneous solution was formed. Correspondingly, the molar ratio for salt: water: [Pyr_{13}][TFSI] is 1:1:9.7. Similarly, with the same contents of water and [Pyr_{13}][TFSI], the mass of LiTFSI was 0.2066 g and 0.4132 g, respectively, to prepare the salt-in-humid IL with the molar salt-water ratio of 2:1 and 4:1. With the same approach, to prepare humid [Bmim][TFSI], 7.7 μL (0.43 mmol) ultrapure water was added into dry [Bmim][TFSI] (1.00 mL, 1.44 g cm^{-3}, 3.43 mmol), and then the mixture was stirred till homogeneous solution was formed. Thus, the molar ratio of water to IL is around 1:8 and the water content was ~5326 ppm, close to 5168 ppm adopted in MD simulation. Meanwhile, 7.7 μL (0.43 mmol) ultrapure water and 0.1234 g (0.43 mmol) LiTFSI were added into 1.00 mL [Bmim][TFSI], and the mixture was stirred to form salt-in-humid IL with the molar salt-water ratio is 1:1. It should be noted that the molar water-IL ratio in humid IL is equal to that in salt-in-humid IL, and the smaller water concentration in salt-in-humid IL is because of the increase in total mass of electrolyte with adding salt. The water content for all electrolytes used is listed in **Supplementary Table 2**.

Supplementary Table 2. The water content for humid ionic liquid (IL) and salt-in-humid IL electrolytes.

		salt-water ratio	water content (ppm)
[Pyr_{13}][TFSI]	Humid IL	0	4474
	Salt-in-humid IL	1:1	4157
		2:1	3895
		4:1	3460
[Bmim][TFSI]	Humid IL	0	5326
	Salt-in-humid IL	1:1	4901

To experimentally check whether the solubility limit of salt in ILs has been reached in our salt-in-humid IL electrolytes, we dissolved LiTFSI into humid [Pyr_{13}][TFSI] and [Bmim][TFSI] with a molar salt-water ratio of 4:1 (the highest salt-water ratio used in this work), and obtained solutions of 0.276LiTFSI-0.069H_2O-0.655[Pyr_{13}][TFSI] and 0.296LiTFSI-0.074H_2O-0.630 [Bmim][TFSI]. Specifically, the melting point of LiTFSI-[Pyr_{13}][TFSI] binary mixture is lower than 21 ℃ when the ratio of salt to IL is lower than 3:7 (i.e., 0.3LiTFSI-0.7[Pyr_{13}][TFSI]).[2] Herein, the salt-IL ratio for salt-in-humid [Pyr_{13}][TFSI] is lower than the that in LiTFSI-[Pyr_{13}][TFSI] binary mixture, indicating that the melting point for salt-in-humid [Pyr_{13}][TFSI] is lower than 21 ℃. Furthermore, **Supplementary Figure 3** shows that no undissolved crystal is observed in the vessel. Therefore, the solubility limit of LiTFSI in the salt-in-humid ILs studied still does not approach.

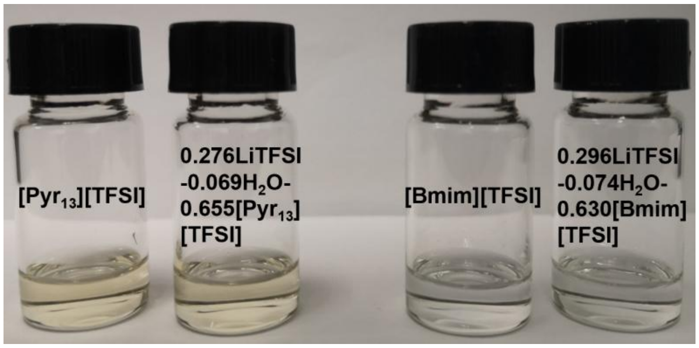

Supplementary Figure 3 | Images of pure ionic liquid and salt-in-humid ionic liquid solutions. No undissolved crystal is observed.

Supplementary Note 3: HOPG surface characterization

The possible defect of highly oriented pyrolytic graphite (HOPG) is detected by scanning tunneling microscope (STM) measurements that were conducted on a scanning probe microscope (Bruker Corp., multimode 8). An obtained STM image is shown in **Supplementary Figure 4**, which exhibits that only one step edge was observed at a scan area of $0.5 \times 0.5\ \mu m^2$. Then the proportion of atom number at the step edge is estimated to be *ca.* 2.1×10^{-4}, indicating that the effect of step edges could be negligible for HOPG used in this work. Therefore, in simulation systems (both MD and DFT), we constructed the atomically flat graphite electrodes without consideration of step edges.

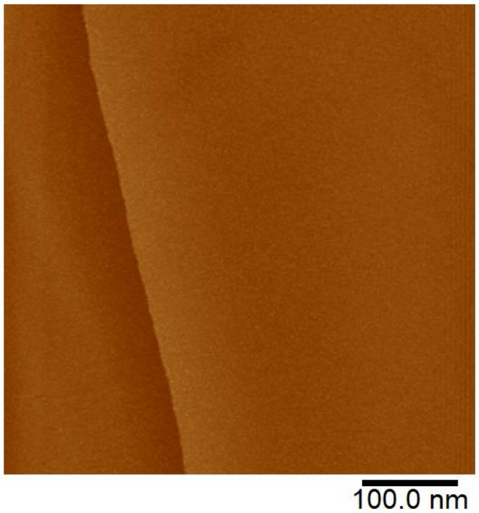

Supplementary Figure 4 | STM image of HOPG electrode surface. The proportion of atom number at the step edge is estimated to be around 2.1×10^{-4}, indicating that the effect of step edges could be negligible in this work.

Supplementary Note 4: Interfacial structure of electrolyte/electrode interfaces

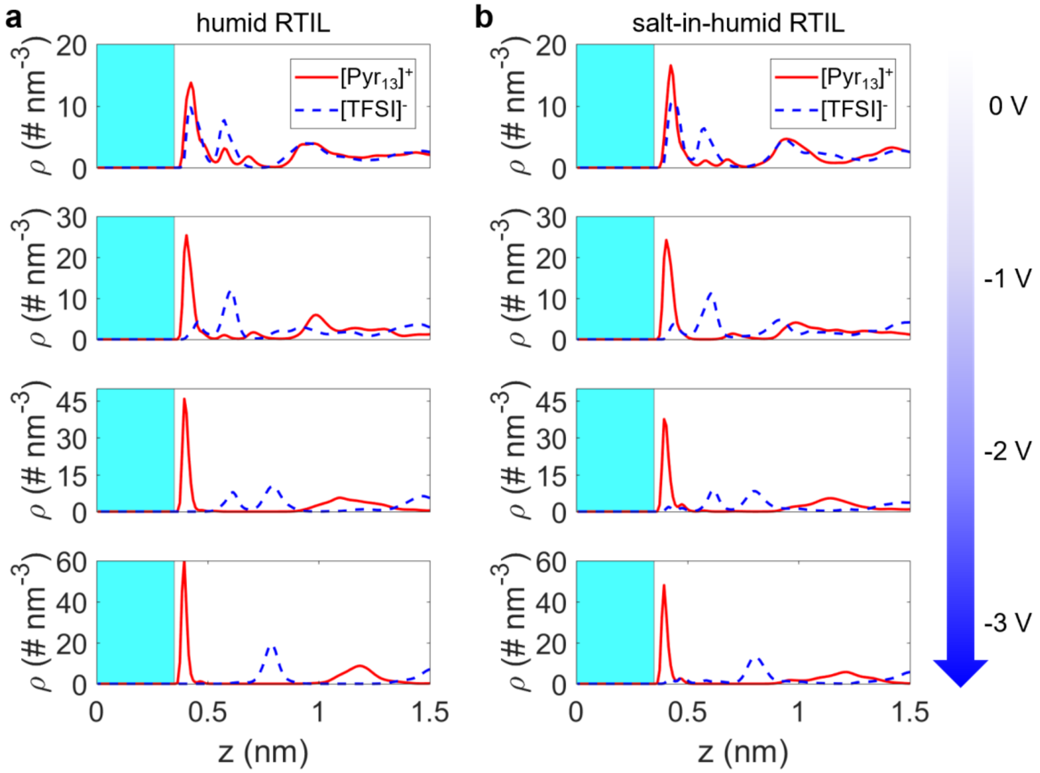

Supplementary Figure 5 | Number density profiles of ionic liquids at neutral and negative electrodes. The number density profiles (ρ) of ionic liquid cation 1-methyl-1-propylpyrrolidinium ($[Pyr_{13}]^+$) and anion bis(trifluoromethylsulfonyl)imide ($[TFSI]^-$) as a function of distance from the electrode surface (z) in humid $[Pyr_{13}]$[TFSI] **(a)** and salt-in-humid $[Pyr_{13}]$[TFSI] electrolyte **(b)**. The cyan shaded region ($z < 0.35$ nm) is considered to be the interfacial region. The graphs from top to bottom represent the applied 0, -1, -2 and -3 V, respectively.

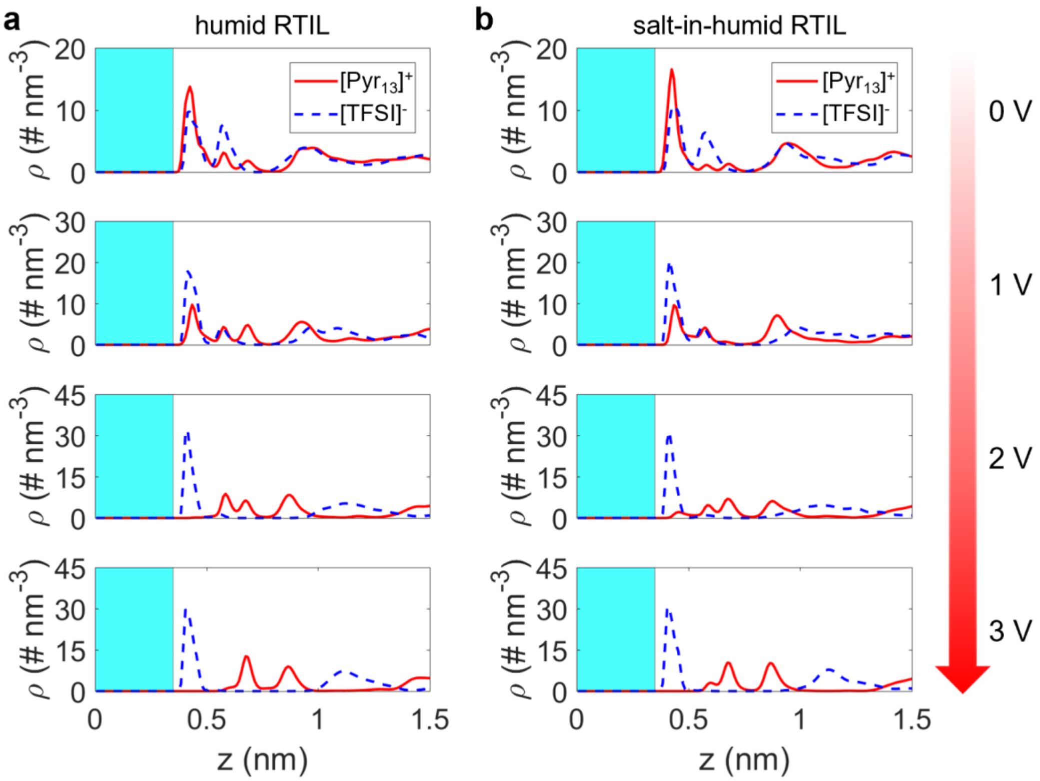

Supplementary Figure 6 | Number density profiles of ionic liquids at neutral and positive electrodes. The number density profiles (ρ) of ionic liquid cation 1-methyl-1-propylpyrrolidinium ($[Pyr_{13}]^+$) and anion bis(trifluoromethylsulfonyl)imide ($[TFSI]^-$) as a function of distance from the electrode surface (z) in humid $[Pyr_{13}][TFSI]$ **(a)** and salt-in-humid $[Pyr_{13}][TFSI]$ electrolyte **(b)**. The cyan shaded region ($z < 0.35$ nm) is considered to be the interfacial region. The graphs from top to bottom represent the applied 0, 1, 2 and 3 V, respectively.

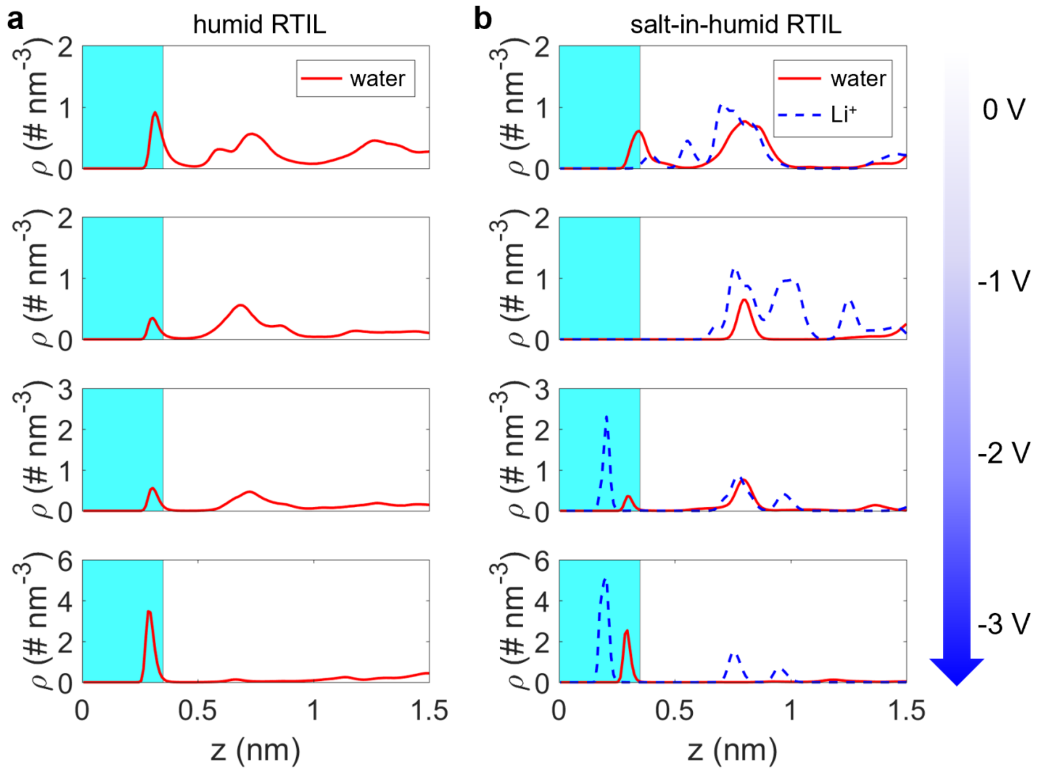

Supplementary Figure 7 | Number density profiles of water and Li^+ at neutral and negative electrodes. The number density profiles (ρ) of water and added Li^+ as a function of distance from the electrode surface (z) in humid 1-methyl-1-propylpyrrolidinium bis(trifluoromethylsulfonyl)imide ([Pyr_{13}][TFSI]) **(a)** and salt-in-humid [Pyr_{13}][TFSI] electrolyte **(b)**. The green cyan region (z < 0.35 nm) is considered to be the interfacial region. The graphs from top to bottom represent the applied 0, -1, -2 and -3 V, respectively.

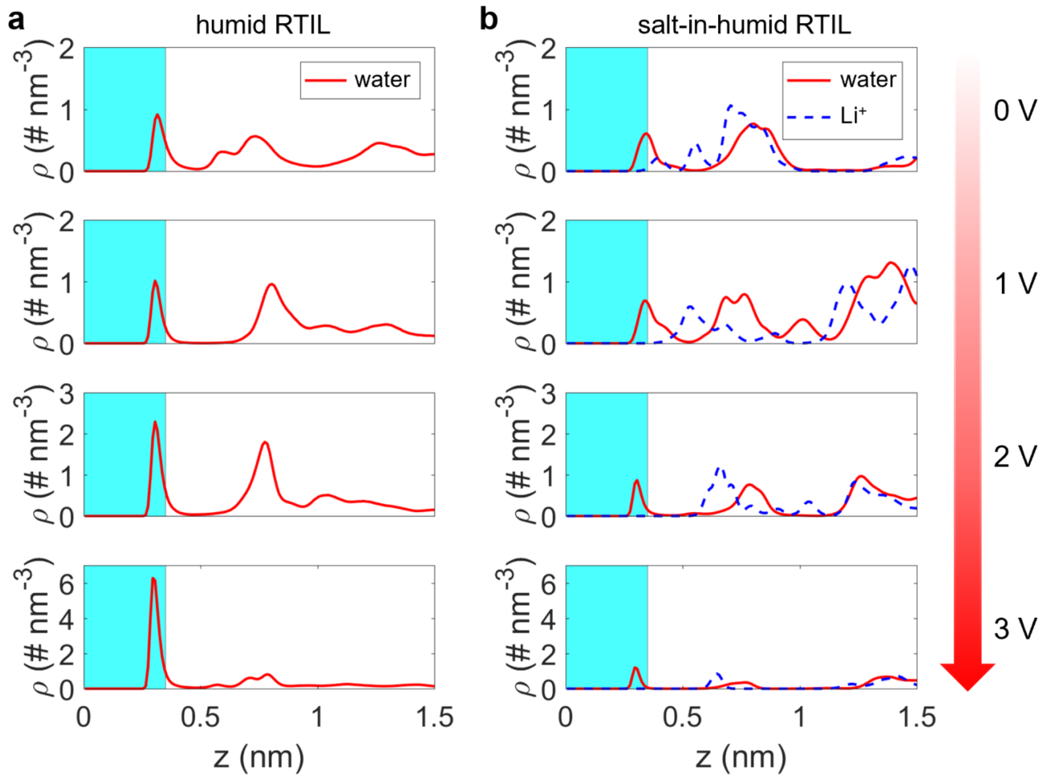

Supplementary Figure 8 | Number density profiles of water and Li^+ at neutral and positive electrodes. The number density profiles (ρ) of water and added Li^+ as a function of distance from the electrode surface (z) in humid [Pyr_{13}][TFSI] **(a)** and salt-in-humid [Pyr_{13}][TFSI] electrolyte **(b)**. The cyan shaded region (z < 0.35 nm) is considered to be the interfacial region. The graphs from top to bottom represent the applied 0, 1, 2 and 3 V, respectively.

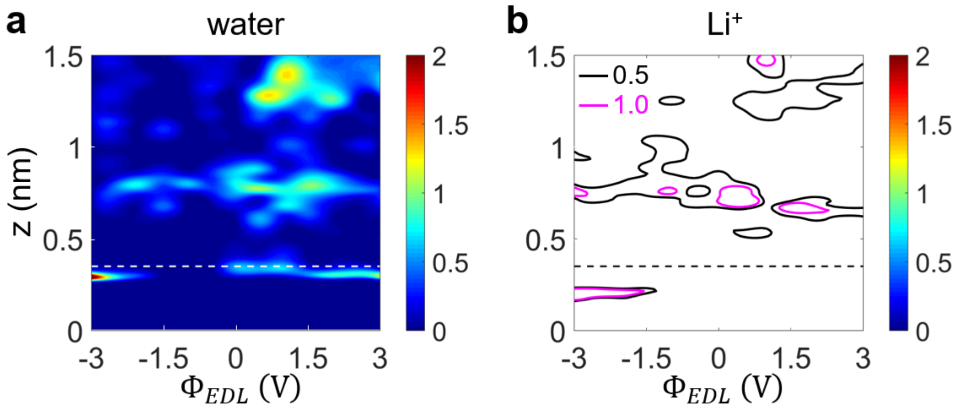

Supplementary Figure 9 | Water and Li^+ distributions under various voltages in salt-in-humid ionic liquids. a-b, Number densities of water (**a**) and Li^+ (**b**) in salt-in-humid 1-methyl-1-propylpyrrolidinium bis(trifluoromethylsulfonyl)imide ([Pyr_{13}][TFSI]). The horizontal dash lines (z = 0.35 nm) in (**a**) and (**b**) represent the upper right boundary of the interfacial region. Unit: # nm^{-3}. The molar salt-water ratio is 1:1.

Supplementary Note 5: Definition of bound water

Supplementary Figure 10 depicts the number density profile of Li^+ ions around water molecules, which was computed from MD simulations of bulk electrolytes with molar salt-water ratios of 0.5, 1, 2 and 4. Similar with previous work[3-5], water can be divided into that in free and bound states, that is, water molecule is considered to be bound, when the water-Li^+ distance is smaller than the radius of the solvation shell (0.25 nm), otherwise, it is labeled as free water.

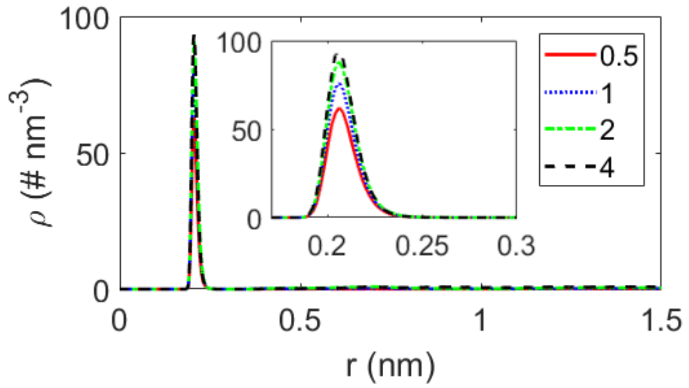

Supplementary Figure 10 | Number density profile of Li^+ ions around water molecule. The number density profile (ρ) of Li^+ ions surrounding water molecules under molar salt-water ratios of 0.5, 1, 2 and 4. Water molecules are considered to be bound to Li^+ within a distance of around 0.25 nm.

Supplementary Note 6: Association of IL and water with interfacial Li^+

We defined the association of interfacial Li^+ with surrounding ionic liquids and water, based on number density profile of Li^+ with anion $[TFSI]^-$ and water from MD simulation of bulk electrolyte with a molar salt-water ratio of 1:1. Herein, Li^+ is considered to be associated with $[TFSI]^-$, when the Li^+-$[TFSI]^-$ distance is smaller than the radius of the solvation shell (the first valley of the number density profile of Li^+-$[TFSI]^-$, see **Supplementary Figure 11a**). It can be seen in **Supplementary Figure 11b** that interfacial Li^+ ions were mainly associated with $[TFSI]^-$, and some interfacial Li^+ ions were associated with water. One snapshot of association of Li^+ with $[TFSI]^-$ and with water at negative electrode (-2 V) is shown in **Supplementary Figure 11c**.

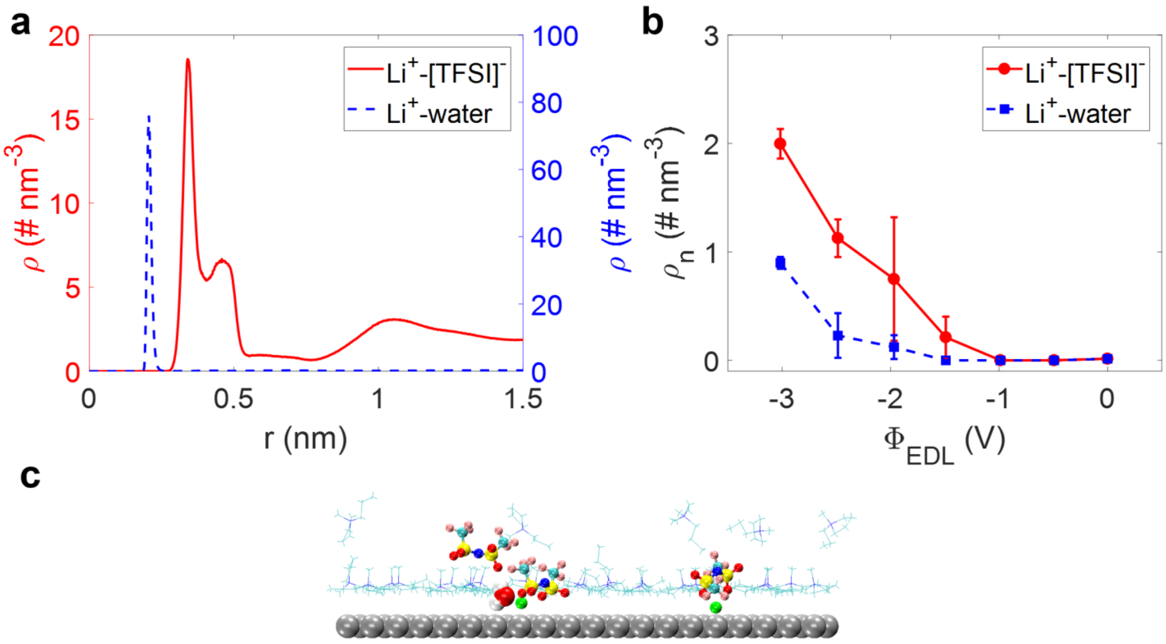

Supplementary Figure 11 | The association of Li^+ with its neighbors. a. The number density profile (ρ) of Li^+ with anion bis(trifluoromethylsulfonyl)imide ($[TFSI]^-$) and with water from bulk molecular dynamics simulation with a molar salt-water ratio of 1:1. **b**. The number density profile (ρ_n) of Li^+ associated with anion $[TFSI]^-$ and with water in the interfacial region. **c.** The snapshot of association of Li^+ with water and with anion $[TFSI]^-$ at the negative electrode (-2 V).

As for the water on negative electrode surface in salt-in-humid IL system (**Fig. 2a** in the main text), water is apt to be pulled away from the electrode surface under moderate negative polarization. Nevertheless, under high voltages, some water molecules can be attracted on the electrode surface. The association of interfacial water with Li^+ is analyzed quantitatively, that is, the proportion of water in bound state is found to increase with the polarization (e.g., 66.38% at -2 V and 99.99% at -3 V, **Supplementary Table 3**), indicating that interfacial water molecules are mostly bound with Li^+.

Supplementary Table 3. Proportion of bound water in salt-in-humid ionic liquid electrolyte in interfacial region at negative electrode.

	-3 V	-2.5 V	-2 V	-1.5 V	-1.0 V	-0.5 V	0 V
Bound water	99.99% ±0.01%	88.31% ±3.36%	66.38% ±10.57%	-	-	-	79.52% ±8.88%

Note: water is depleted in the interfacial region under negative polarization of -1.5 ~ -0.5 V.

Supplementary Note 7: OCP and scan rate effect

Supplementary Figure 12 depicts the time-dependence of the open-circuit potential (OCP) of Ag quasi-reference electrode vs. Ag/AgCl reference electrode in different electrolytes. The OCP of Ag quasi-reference electrode fluctuated a little bit, and the OCP and error bar in different electrolytes were listed in **Supplementary Table 4**. Subsequently, CVs have been corrected accordingly based on the obtained OCPs.

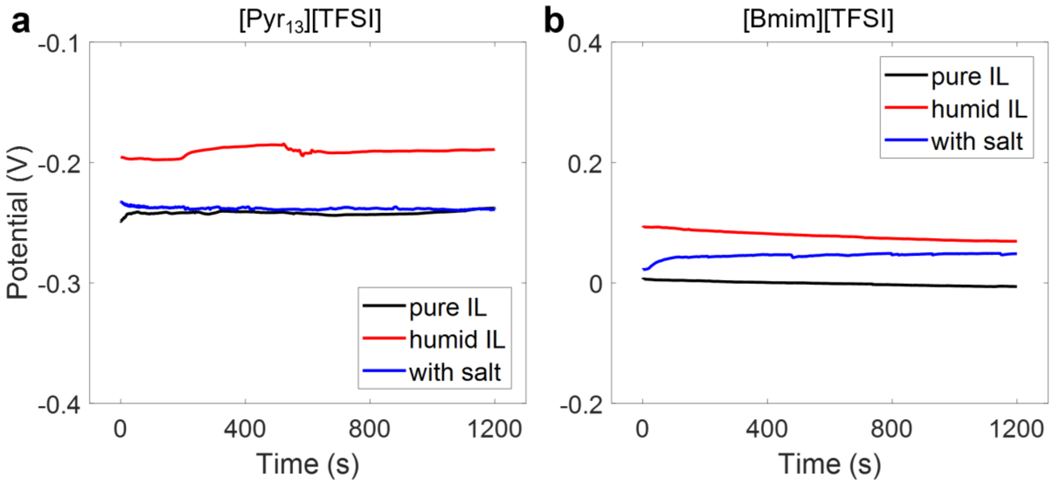

Supplementary Figure 12 | Time-dependence of open-circuit potential. Time-dependence of open-circuit potential (OCP) of Ag quasi-reference electrode vs. Ag/AgCl electrode in 1-methyl-1-propylpyrrolidinium bis(trifluoromethylsulfonyl)imide ([Pyr_{13}][TFSI]), humid [Pyr_{13}][TFSI] and salt-in-humid [Pyr_{13}][TFSI] (**a**), and in 1-butyl-3-methylimidazolium TFSI ([Bmim][TFSI]), humid [Bmim][TFSI] and salt-in-humid [Bmim][TFSI] (**b**).

Supplementary Table 4. The open-circuit potential (OCP) and error of Ag wire versus the Ag/AgCl reference electrode in different electrolytes. Unit: V

	pure IL	humid IL	with salt
[Pyr_{13}][TFSI]	-0.24±0.03	-0.19±0.05	-0.24±0.04
[Bmim][TFSI]	0.00±0.04	0.08±0.05	0.03±0.04

To testify the effect of scan rate on the electrochemical window, we carried out additional CV measurements for pure [Pyr_{13}][TFSI], humid [Pyr_{13}][TFSI], and salt-in-humid [Pyr_{13}][TFSI]. The water content for humid [Pyr_{13}][TFSI] is ~4594 ppm, and molar salt-water ratio is 1:1 for salt-in-humid [Pyr_{13}][TFSI] that has the same molar water-IL ratio with humid [Pyr_{13}][TFSI]. With scan rates of 5, 10, 50 and 100 mV s^{-1}, cyclic voltammograms of HOPG in three types of electrolytes were shown in **Supplementary Figure 13**. It can be seen that as the scan rate is reduced from 100 to 5 mV s^{-1}, the electrochemical window changes very little, so that the phenomenon that the electrochemical window of humid IL could be enhanced by adding salt is unchanged with varying the scan rate of CV measurements.

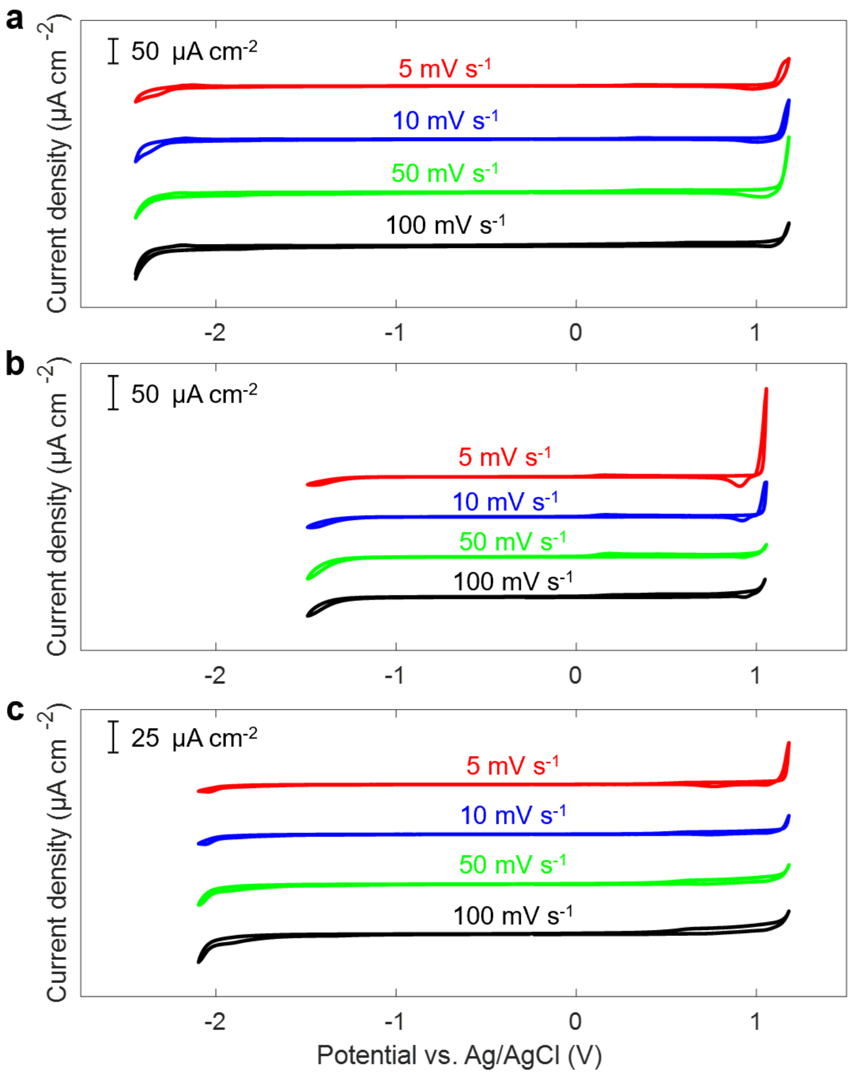

Supplementary Figure 13 | Cyclic voltammograms of HOPG with electrolytes at different scan rates. **a-c,** Cyclic voltammogram (CV) profiles of HOPG with pure 1-methyl-1-propylpyrrolidinium bis(trifluoromethylsulfonyl)imide ($[Pyr_{13}][TFSI]$) (**a**), humid $[Pyr_{13}][TFSI]$ (**b**) and salt-in-humid $[Pyr_{13}][TFSI]$ (**c**) at different scan rates (5, 10, 50 and 100 mV s^{-1}). The water content for humid $[Pyr_{13}][TFSI]$ is ~4594 ppm. The molar salt-water ratio for salt-in-humid $[Pyr_{13}][TFSI]$ electrolyte is 1:1.

Supplementary Note 8: Effect of the molar salt-water ratio

The effect of molar salt-water ratio on water adsorption and the electrochemical window was investigated, via both MD simulation and CV measurements. With different molar salt-water ratios (1:1 and 2:1), revealed by MD simulation, the amount of water adsorption exhibits the same trend with adding more salt, and the interfacial water is even excluded more notably (**Supplementary Figure 14a**); meanwhile, the CV measurements show the nearly same expansion of the electrochemical window compared with that of humid IL (**Supplementary Figure 14b**).

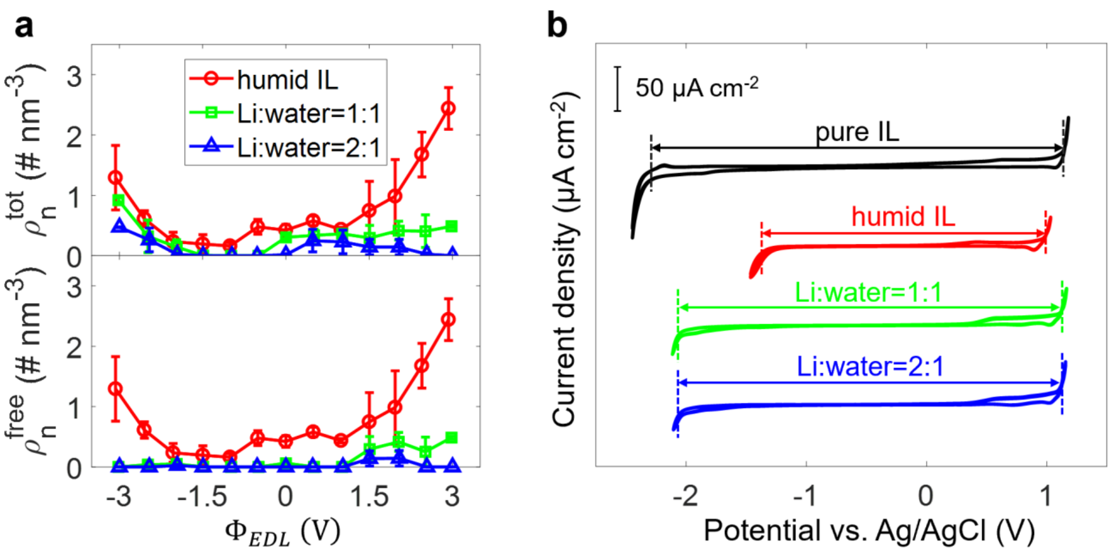

Supplementary Figure 14 | Effect of adding salt on water adsorption near electrode surface and the electrochemical window. **a,** Electrosorption of water from humid 1-methyl-1-propylpyrrolidinium bis(trifluoromethylsulfonyl)imide ([Pyr_{13}][TFSI]) and salt-in-humid [Pyr_{13}][TFSI] electrolyte on electrodes. The top and bottom panels are the number densities of total water (ρ_n^{tot}) and free water (ρ_n^{free}) respectively. **b,** Cyclic voltammograms (CV) of HOPG with pure [Pyr_{13}][TFSI], humid [Pyr_{13}][TFSI] and salt-in-humid [Pyr_{13}][TFSI] electrolyte. Scan rate: 100 mV s^{-1}. Water contents for humid ILs are around 4392 and 4474 ppm, for MD simulations and CV experiments, respectively.

Supplementary Note 9: IR spectrum and H-bond of water

9.1 MD simulation

The total dipole moment, $\boldsymbol{M}(t)$, of the system at any time, t, is calculated as:

$$\boldsymbol{M}(t) = \sum_{i=1}^{n} e_i \boldsymbol{r}_i(t) \quad (1)$$

where n is the total number of atoms, e_i and $\boldsymbol{r}_i$ are the charge and the position vector of the ith atom. The infrared (IR) spectrum in equilibrium is obtained by Fourier transforming the dipole moment autocorrelation function[6-9], which is described as:

$$I(\omega) \propto \int_0^{\infty} \langle \boldsymbol{M}(t)\boldsymbol{M}(0)\rangle cos(\omega t)dt \quad (2)$$

where $I(\omega)$ is the spectral density, and ω is the angular frequency of the IR radiation. Using Parseval's theorem[10], the Wiener-Khintchine theorem[10], and the properties of Fourier transforms, the IR spectrum, in periodic boundary conditions, can be converted to the time derivative autocorrelation function as[9,10],

$$\mathrm{I}(\omega) \propto \int_0^{\infty} \langle \frac{d\boldsymbol{M}(t)}{dt}\frac{d\boldsymbol{M}(0)}{dt}\rangle\, cos(\omega t)dt = \int_0^{\infty} \langle \sum_{j=1}^{n} e_j \boldsymbol{v}_j(t) \sum_{j=1}^{n} e_j \boldsymbol{v}_j(0)\rangle\, cos(\omega t)dt \quad (3)$$

9.2 Experiment

IR measurements were also carried out to experimentally explore the O-H stretching vibration. **Supplementary Figure 15** exhibits the IR spectra of water in humid IL and salt-in-humid IL electrolytes. Three Lorentz peak functions are taken to decompose the spectrum of O-H stretching vibration, with each peak representing one type of water[11,12]. The red, pink and blue regions represent three different types of water in electrolyte. It is common and expected that the peak position of predicted vibration position has some deviation from the experimental result.[13] Therefore, in order to better understand the IR spectra, we, herein, concentrate on the shift of the stretching vibration rather than the absolute value. Taking the peak position of stretching vibration of water in humid IL as reference, the comparison of peak shifts of IR spectra from MD simulation and experiment has been plotted in **Supplementary Figure 16**, which reveals that, despite the numerical differences, the blue-shifts occur with adding salt in both MD simulation and experiment.

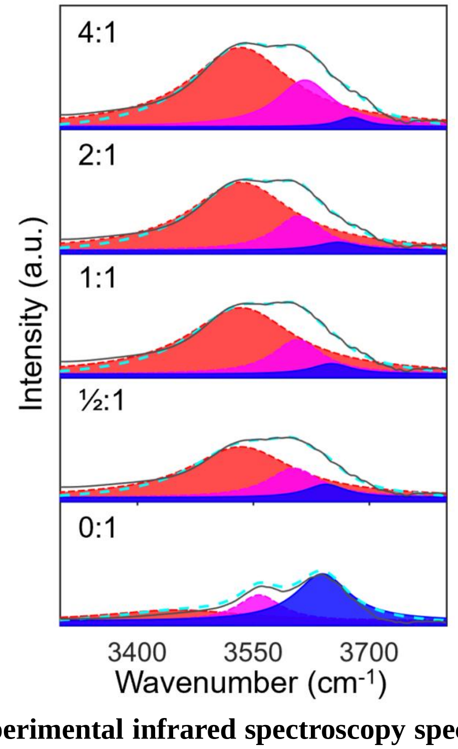

Supplementary Figure 15 | Experimental infrared spectroscopy spectra. They are for water in humid 1-methyl-1-propylpyrrolidinium bis(trifluoromethylsulfonyl)imide ([Pyr_{13}][TFSI]) and in humid [Pyr_{13}][TFSI] with different molar salt-water ratios (from 0.5 to 4). The red, pink and blue regions are the first, second and third fitting spectra, respectively, each representing one type of water. The cyan dash lines and gray solid lines represent the summation of the fitting and experimental spectra, respectively.

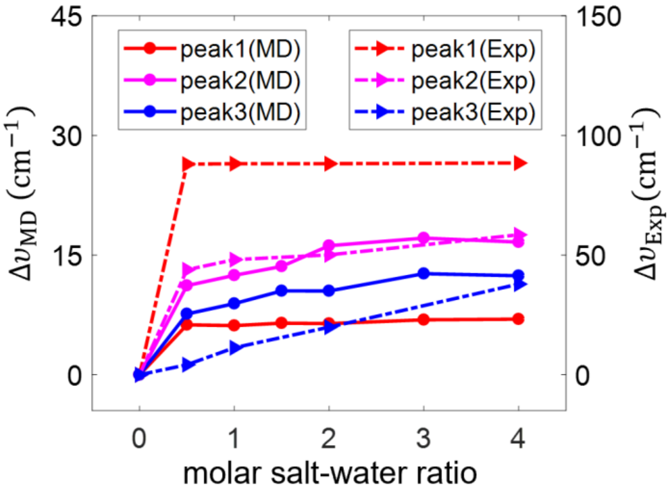

Supplementary Figure 16 | Comparison of simulation-calculated and experimental peak position shifts of infrared spectroscopy spectra. The spectrum of O-H stretching vibration is assigned into three different models. Taking the peak position of O-H stretching vibration of water in humid ionic liquid (IL) as reference, $\Delta\upsilon$ is the shift of peak position of O-H stretching vibration of water in humid IL with adding salt.

9.3 DFT calculations

We further investigate the IR spectra of water in humid IL and in salt-in-humid IL with B3LYP/DFT calculation using Gaussian 09 program (Revision D.01).[14] Specifically, the vibrational frequency scaling factor is 0.967. Comparing **Supplementary Figure 17a** where water cluster is associated with IL (Each O-H bond of water forms an H-bond with its neighbors, including water-water and water-IL) with **Supplementary Figure 17b** where one molecule is associated with IL (each O-H bond of water can form an H-bond between water and IL), the peak position of O-H stretching vibrational of water is found to shift towards higher wavenumber. Furthermore, an even higher wavenumber of peak position of stretching vibration occurs in the presence of free O-H bond without H-bond (**Supplementary Figure 17c**). These phenomena indicate that breaking hydrogen bond of water molecules can lead to the higher wavenumber. Moreover, when water is associated with Li^+ in IL environment, an even higher wavenumber occurs (**Supplementary Figure 17d-f**).

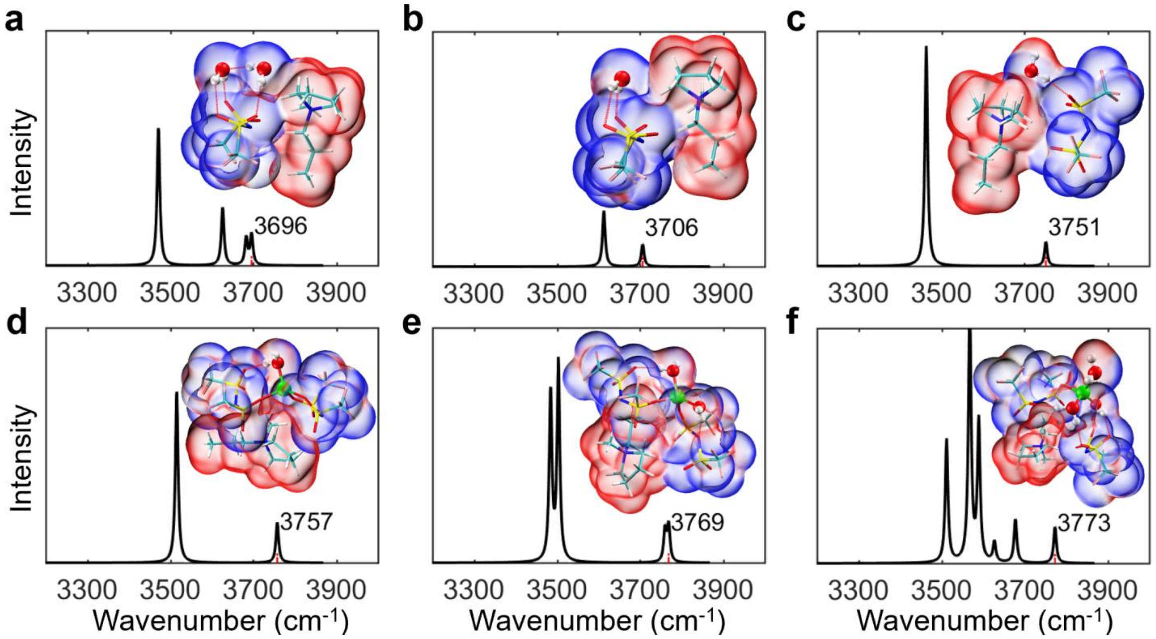

Supplementary Figure 17 | The infrared spectroscopy spectrum of ionic liquid/water/Li[TFSI] clusters. a-c, The infrared spectroscopy (IR) spectrum of IL/water clusters based on DFT calculation.[15] **a**, water cluster (two water molecules) associated with IL pair. Each O-H bond of water forms an H-bond with its neighbors, including water-water and water-IL. **b**, one water molecule associated with IL pair. Each O-H bond of water can form an H-bond between water-IL. **c**, one water molecule associated with IL. The water molecule forms an H-bond with IL, remaining a free O-H bond. **d-f,** The IR spectrum of water in $Li^+(H_2O)$ (**d**), $Li^+(H_2O)_2$ (**e**) and $Li^+(H_2O)_3$ (**f**) associated with IL pair.

9.4 H-bond

We analyzed the number of H-bonds that the water molecule could form with surrounding water molecules/cations/anions. Herein, to define the H-bond, a geometrical criterion is used (**Supplementary Figure 18a**) as $r_{HB} \leq 0.35\,nm$ and $\alpha_{HB} \leq 30^{\circ}$,[16,17] where r_{HB} is the distance between the possible donor with a hydrogen (H) bonded to it, and acceptor which is not bonded to donor, and α_{HB} is the angle of hydrogen-donor-acceptor.

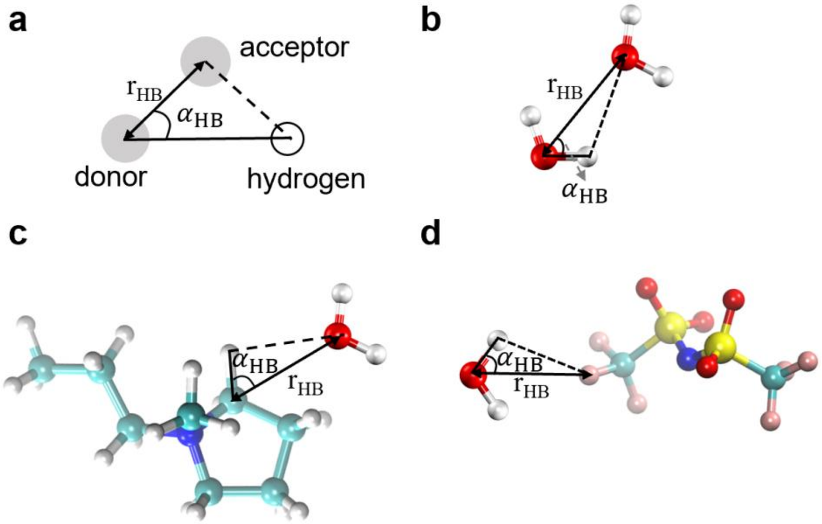

Supplementary Figure 18 | Geometrical hydrogen bond criterion. a, The schematics of geometrical criterion for the hydrogen bond. r_{HB} is the distance between the donor and acceptor, and α_{HB} is the angle of hydrogen-donor-acceptor. **b-d,** The schematics of H-bond by water molecules with water molecules, cations and anions in 1-methyl-1-propylpyrrolidinium bis(trifluoromethylsulfonyl)imide ([Pyr_{13}][TFSI]).

Specifically, for H-bond between water and water (**Supplementary Figure 18b**), we define the H-bond between a hydrogen atom of water and an oxygen atom of another water to be formed, if the distance between the two oxygen atoms is shorter than or equal to 0.35 nm and the angle of hydrogen-oxygen-oxygen is less than or equal to 30 °. H-bonds between water and ILs can be seen in **Supplementary Figure 18c-d**. For a cation, H-bond can be defined between water and hydrogen of cation; for an anion, H-bonds are determined between water and an electronegative atoms of anion (acting as acceptor, here are fluorine, nitrogen or oxygen atom).

9.5 Water association with Li^+ in bulk electrolyte

Supplementary Figure 19 presents the water cluster or hydrated Li^+ cluster in humid [Pyr_{13}][TFSI] and salt-in-humid [Pyr_{13}][TFSI]. The cluster is determined based on the corresponding hydration shell. In humid IL, there are some water clusters (e.g., $(H_2O)_2$, $(H_2O)_3$, $(H_2O)_4$, and $(H_2O)_5$, see **Supplementary Figure 19a**). However, with the addition of Li^+ ions, water molecules tend to associate with Li^+ to form "bound" water, resulting in the reduction of single free water molecule and the complete disappearance of the water clusters (**Supplementary Figure 19b-c**). Meanwhile, as shown in **Supplementary Figure 20**, the coordination number of Li^+ ions surrounding a water molecule increases from about 0.61 to 0.95 as the molar salt-water ratio varies from 0.5:1 to 4:1.

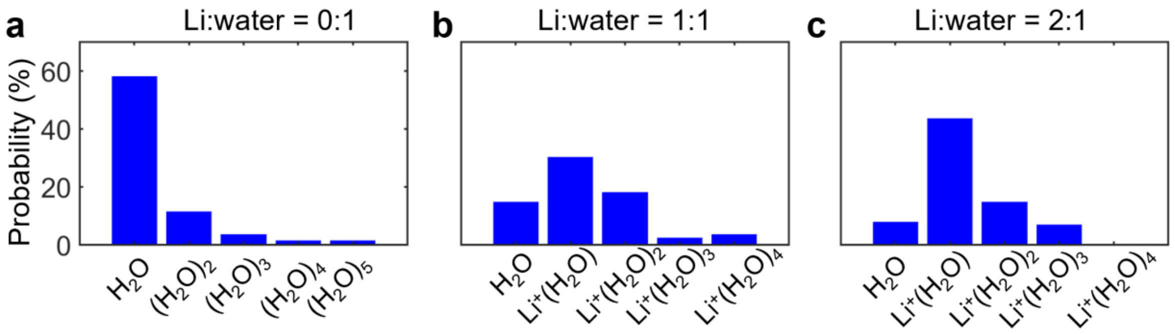

Supplementary Figure 19 | Water cluster in different electrolytes. a, The cluster distribution of water in humid 1-methyl-1-propylpyrrolidinium bis(trifluoromethylsulfonyl)imide ([Pyr_{13}][TFSI]). **b-c**, The cluster distribution of water and its association with Li^+ when the molar salt-water ratios are 1:1 (**b**) and 2:1 (**c**).

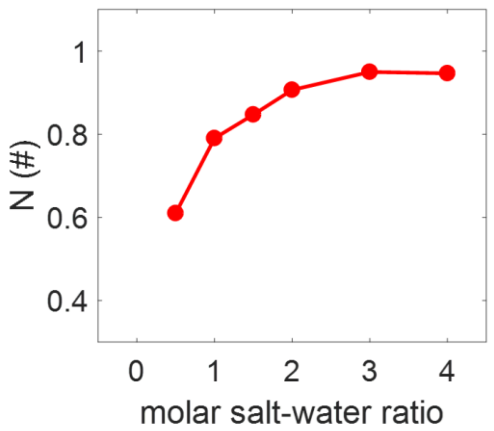

Supplementary Figure 20 | Coordination number of Li^+ associated with water. The coordination number of Li^+ associated with water within the hydration shell under varied molar salt-water ratios from bulk molecular dynamics simulation. The coordination number of Li^+ around water increases with adding salt.

Supplementary Note 10: Umbrella sampling method

When using umbrella sampling method to calculate PMF, a series of initial configurations are generated, and each corresponds to a location where the molecule is restrained via a harmonic potential.[18] Taking water as an example, it could be understood as the water was bound by a spring: water could move back and forward around a given position along the direction vertical to the electrode surface, and travel freely in the plane parallel to the electrode surface. For instance, 48 different configurations of water, shown in **Supplementary Figure 21a**, were constructed. With the weighted histogram analysis method[18], the PMF curve of water at -2 V (**Fig. 4a**) was obtained by performing MD simulations with 48 different configurations. In simulation, the water molecule, rather than being fixed, is allowed to rotate, interacting with its surroundings and thus experiencing all possible orientations. The schematic of orientation distribution of water molecule is shown in **Supplementary Figure 21b**, where θ_{dipole} is defined as the angle between the normal of electrode surface and the water vector, and θ_{normal} is the angle formed between the normal of electrode surface and the normal of water plane. As seen in **Supplementary Figure 21c-f**, the orientations of water molecule at several typical sampling points (0.29, 0.47, 0.75 and 2.35 nm) are calculated. It can be seen that the orientation distributions at these four sampling points are quite different from each other. In particular, the orientation distribution of water molecule at ~0.29 nm shows that the water is much more ordered than that at ~2.35 nm, and the later water is far from the electrode surface, consistent with that in the bulk humid ionic liquids.

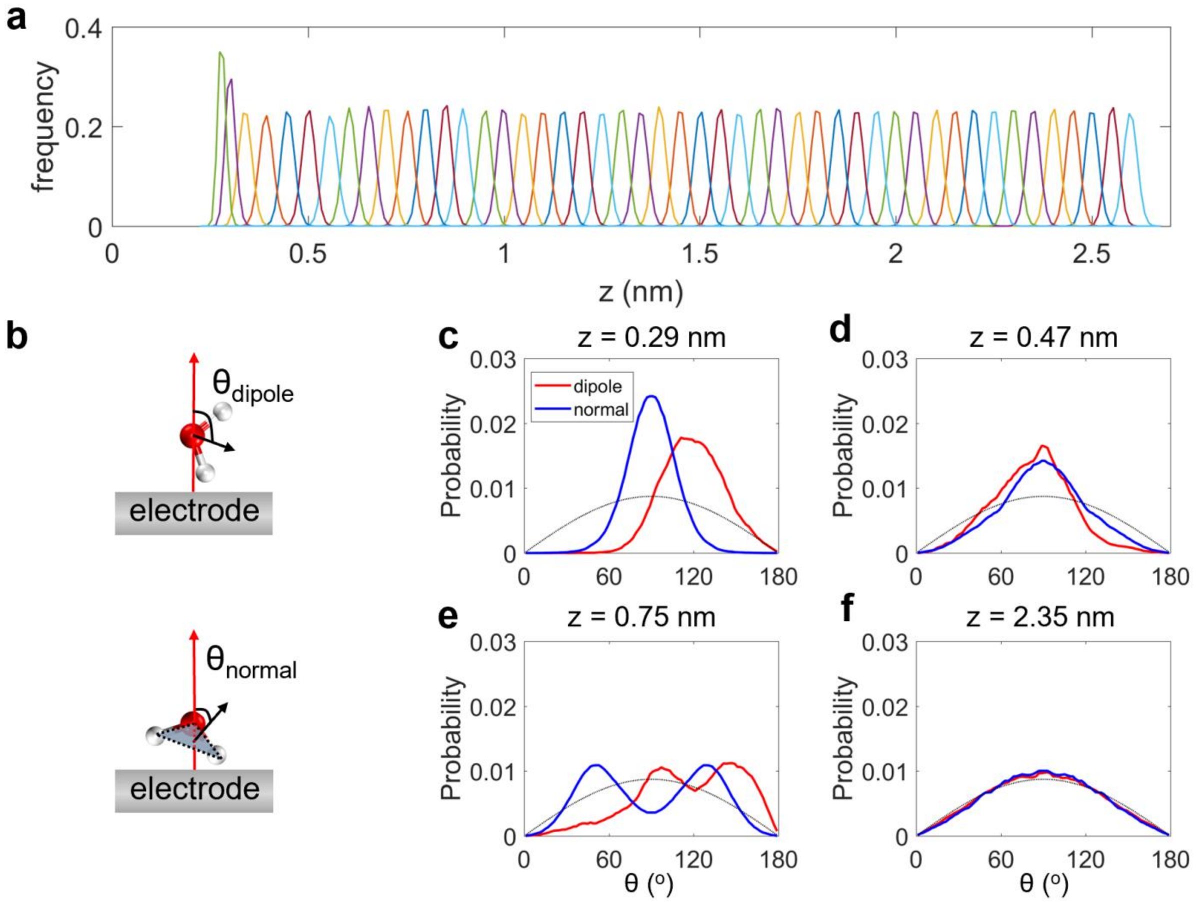

Supplementary Figure 21 | Umbrella sampling method to calculate the potential of mean force information of water. a, Weights of weighted histogram analysis method with 48 configurations of molecular dynamics simulations. **b**, The schematic of orientation distribution of water molecule. θ_{dipole} is defined as the angle between the normal of electrode surface and the water vector, and θ_{normal} is the angle formed between the normal of electrode surface and the normal of water plane. **c-f**, The orientation distribution of water molecule at corresponding sampling window, i.e., 0.29 nm (**c**), 0.47 nm (**d**), 0.75 nm (**e**), and 2.35 nm (**f**). Red and blue lines in **(c-f)** represent the dipole and normal orientation. The black dotted lines in **(c-f)** represent the orientation of bulk water.

Supplementary Note 11: Arrangement of interfacial water

The atomic densities (oxygen and hydrogen atoms) and orientation distributions of water at several more key potentials (-2.5, -1, -0.5, 0.5 and 2 V) are shown in **Supplementary Figure 22**.

Under negative polarizations, in humid IL, interfacial water adopts a configuration with one O-H bond pointing to the electrode, since the first peak of hydrogen is closer to the surface than that of oxygen (**Supplementary Figure 22a-b**); meanwhile, both dipole and normal orientations of interfacial water change from random distribution to ordered one (**Supplementary Figure 22c-d**). With adding salt, since water is depleted at -0.5 and -1 V, there is no orientation distribution of interfacial water. For -2 V (**Fig. 5** in main text) and -2.5 V, the dipole orientation shifts towards small angle (**Supplementary Figure 22c**), while the normal orientation of interfacial water changes little (**Supplementary Figure 22d**), suggesting that the water is tilted to make hydrogen atom staying further away from the surface (**Supplementary Figure 22b**).

Under positive polarization, in humid IL, the water adopts a configuration nearly parallel to the electrode surface; meanwhile, the second peak for hydrogen atom gradually disappears (**Supplementary Figure 22a-b**). These indicate that the orientation distribution of interfacial water becomes more ordered with the increase of the positive polarization (**Supplementary Figure 22c-d**). After adding salt, the interfacial water at 0.5 and 1 V (**Fig. 5** in main text) is re-arranged to make oxygen staying further away from electrode surface due to the association of Li^+ (**Supplementary Figure 22a-b**); while at 2 V, no water is associated with Li^+, since Li^+ is pushed away from electrode surface, and thus the interfacial water adopts very similar arrangement in humid IL and salt-in-humid IL.

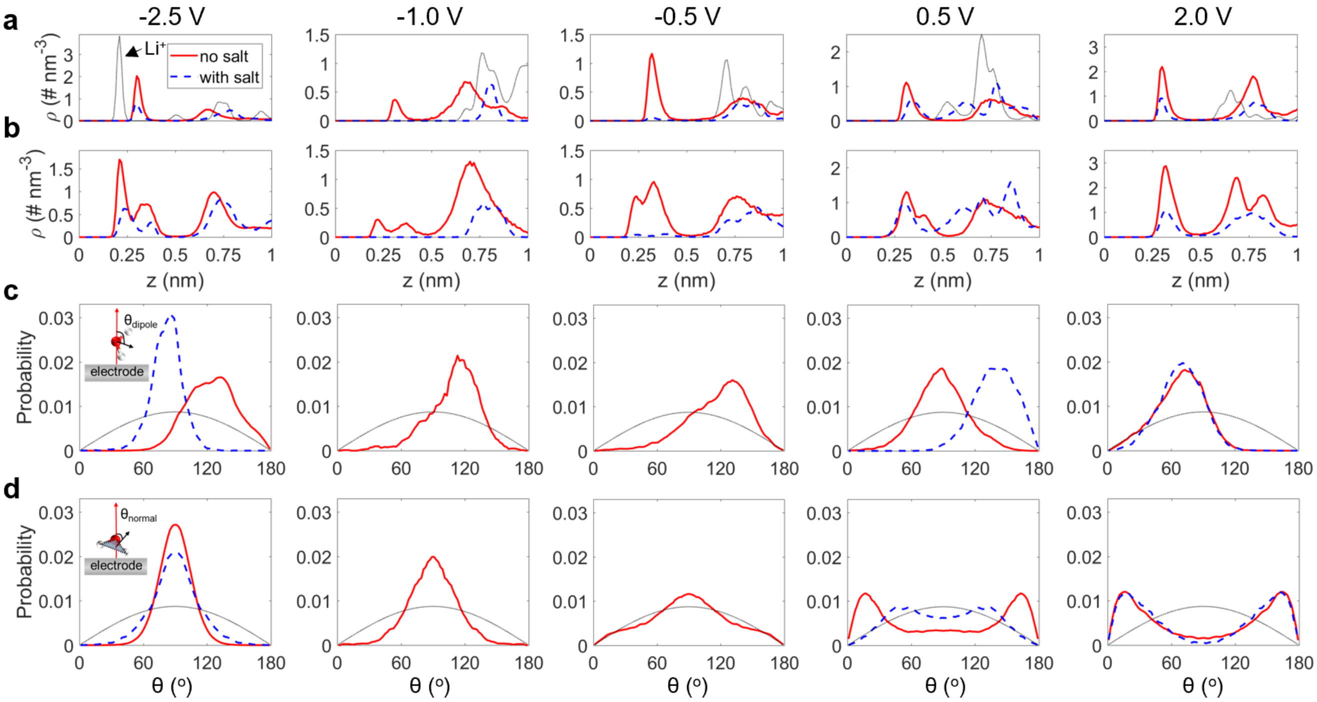

Supplementary Figure 22 | Effect of adding salt on the interfacial water of humid ionic liquid at the electrified surface. a-b, The atomic number densities (ρ) of oxygen (**a**) and hydrogen (**b**) of water in humid ionic liquid (IL) (red solid line) and in salt-in-humid IL (blue dash line) at electrodes. The black dotted line in (**a)** is the atom number density of Li^+ in salt-in-humid IL. **c-d**, The dipole orientation (**c**) and normal orientation (**d**) of interfacial water in humid IL (red solid line) and in salt-in-humid IL (blue dash line) at electrodes. The black dotted line in (**c-d)** represents the orientation of bulk water.

To understand how the water arrangements affect water electrolysis (i.e., possible charge transfer between interfacial water and the electrode), four typical water arrangements were taken from MD simulations (under -2 V and +1 V, before and after adding salt, see **Fig. 5e-f** in main text) to construct the configurations for DFT calculations. These four different water arrangements are chosen as: (1) water in humid IL at -2 V (arrangement-1), (2) water in salt-in-humid IL at -2 V (arrangement-2), (3) water in humid IL at 1 V (arrangement-3), and (4) water in salt-in-humid IL at 1 V (arrangement-4); their corresponding orientations and atom positions listed in **Supplementary Table 5**. The graphite electrode was represented by a 6×8 graphene periodic supercell containing 48 carbon atoms with 20 Å vacuum.

Supplementary Table 5 | The orientation and the position of oxygen and hydrogen atoms of water molecule with respect to the electrode surface.

	negative polarization (-2 V)		positive polarization (1 V)	
	no salt	adding salt	no salt	adding salt
label	arrangement-1	arrangement-2	arrangement-3	arrangement-4
θ_{dipole}	111°	70°	90°	110°
θ_{normal}	90°	90°	15°/165°	20°/160°
O	0.310 nm	0.300 nm	0.300 nm	0.340 nm
H1	0.217 nm	0.244 nm	0.280 nm	0.314 nm
H2	0.358 nm	0.390 nm	0.320 nm	0.340 nm

For each water arrangement, we consider four different adsorption sites (shown in **Supplementary Figure 23**), as general setup for water on graphene[19]. That is, the water is on the top of two adjacent carbon atoms (sites T1 and T2), the center of a carbon-carbon bond (site B), and the hexagon center (site C). For arrangement-1 and arrangement-2, the hydrogen atom, closer to the surface, is on top of the adsorption site; for arrangement-3 and arrangement-4, the oxygen atom is on top of the adsorption site. DFT calculations with differently arranged water adsorbed on graphite electrode were performed, using Vienna ab-initio simulation package (VASP).[20] The Perdew-Burke-Ernzerhof (PBE) exchange-correlation functions of generalized gradient approximation (GGA) were employed in DFT calculations[21]. The projector augmented wave (PAW)[22] method with a cutoff energy of 400 eV was used to describe the interaction between nuclei and electrons. The convergence of energy was employed as 10^{-4} eV. The dipole correction and spin polarization were added for the calculations. The Γ-centered k-point meshes of $3\times3\times1$ were adopted. Additionally, for a correct treatment of physisorption interaction[23], the Grimme's D3 dispersion correction[19] was employed in DFT calculations. The electron transferred between the water molecule and graphite is calculated by means of the DDEC6 charge analysis[24-26].

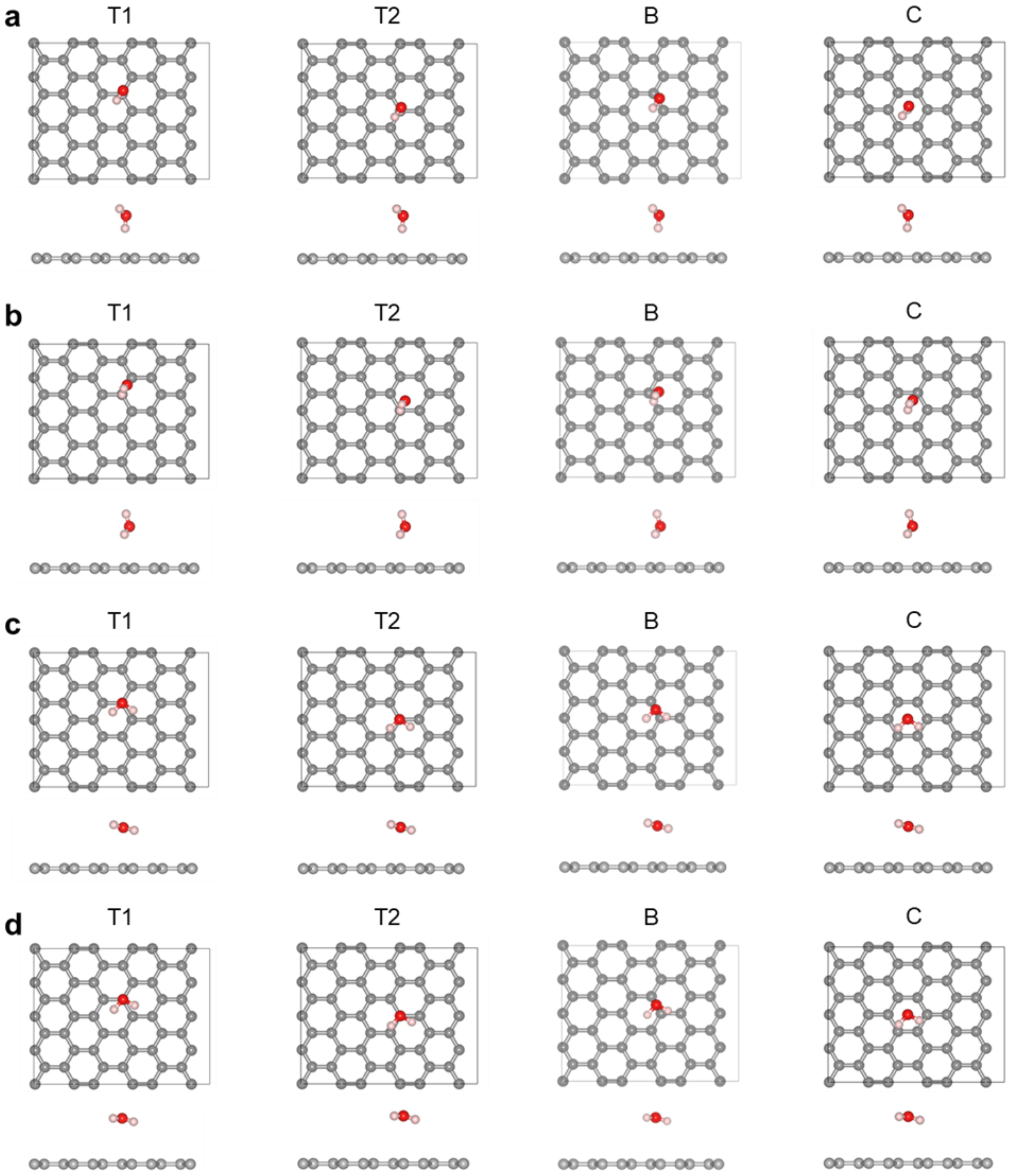

Supplementary Figure 23 | Arrangement of water molecule adsorbed on graphite. a-b, The structures of water molecule adsorbed on graphite on four different adsorption sites with arrangement-1 (**a**) and arrangement-2 (**b**). The hydrogen atom of water, closer to graphite, is on top of four different adsorption sites, i.e. carbon atoms (sites T1 and T2), the center of a carbon-carbon bond (site B), and hexagon center (site C). **c-d**, The structures of water molecule adsorbed on graphite on four different adsorption sites with arrangement-3 (**c**) and arrangement-4 (**d**). The oxygen atom of water is on top of four different adsorption sites, i.e. carbon atoms (sites T1 and T2), the center of a carbon-carbon bond (site B), and hexagon center (site C).

Based on the DDEC6 charge analysis[24-26], the charge transferred between water molecule and graphite is evaluated. As shown in **Supplementary Figure 24a**, the arrangement change due to adding salt (from arrangement-1 to arrangement-2) leads to a reduction of electrons transferred from electrode to water, making it difficult for the hydrogen evolution reaction to take place. Meanwhile, compared with arrangement-3, water molecule in arrangement-4 has fewer positive charges, indicating that adding salt will make water molecule likely lose fewer electrons to the electrode (**Supplementary Figure 24b**), facilitating the inhibition of water oxidation under positive polarization.

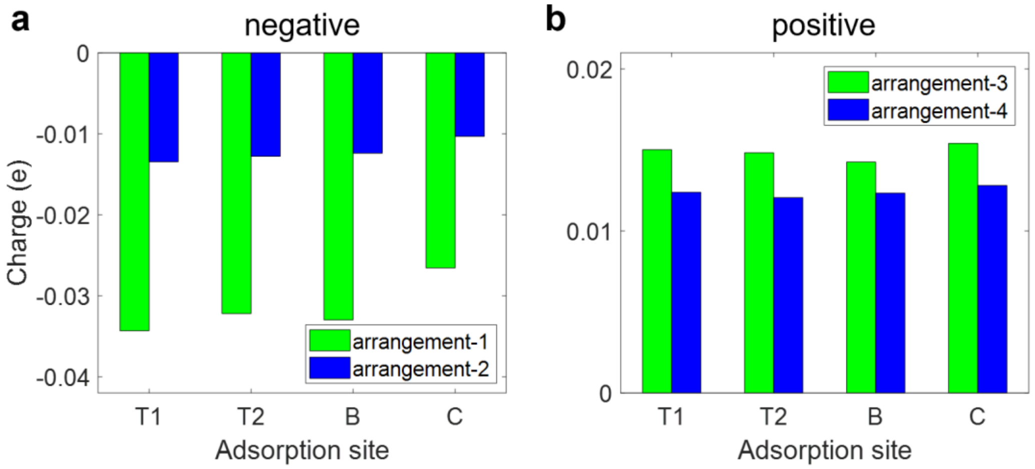

Supplementary Figure 24 | Net charge of water molecule. The net charge on water molecule with different arrangement near negative (a) and positive (b) electrodes.

Supplementary Note 12: Validation by more ionic liquids

To examine the effects of IL type on the findings revealed by both MD simulations and electrochemical measurements of [Pyr_{13}][TFSI], another hydrophobic IL [Bmim][TFSI] is selected (**Supplementary Figure 25a**). The water contents for humid [Bmim][TFSI] are about 5168 and 5326 ppm, for MD simulations and CV measurements, respectively. Meanwhile, the molar salt-water ratio of salt-in-humid-[Bmim][TFSI] electrolyte is 1:1. Detailed information can be seen in **Supplementary Table 1**. In MD simulation, we investigated water electrosorption with varying EDL potential across electrolyte-electrode interface (**Supplementary Figure 25b**). Similar with hydrophobic [Pyr_{13}][TFSI] (**Fig. 2** in main text), it can be found that: 1) for humid [Bmim][TFSI], water preferentially accumulates at the charged electrode surface; 2) after adding salt, interfacial water molecules are found to decrease notably. CV measurements were carried out for pure [Bmim][TFSI], humid [Bmim][TFSI] and salt-in-humid [Bmim][TFSI] with HOPG electrodes (**Supplementary Figure 25c**). When [Bmim][TFSI] becomes humid (~5326 ppm), its electrochemical window is obviously narrowed down. However, after adding salt (the molar salt-water ratio is 1:1), the electrochemical window is clearly widened, in consistent with the observation for salt-in-humid [Pyr_{13}][TFSI].

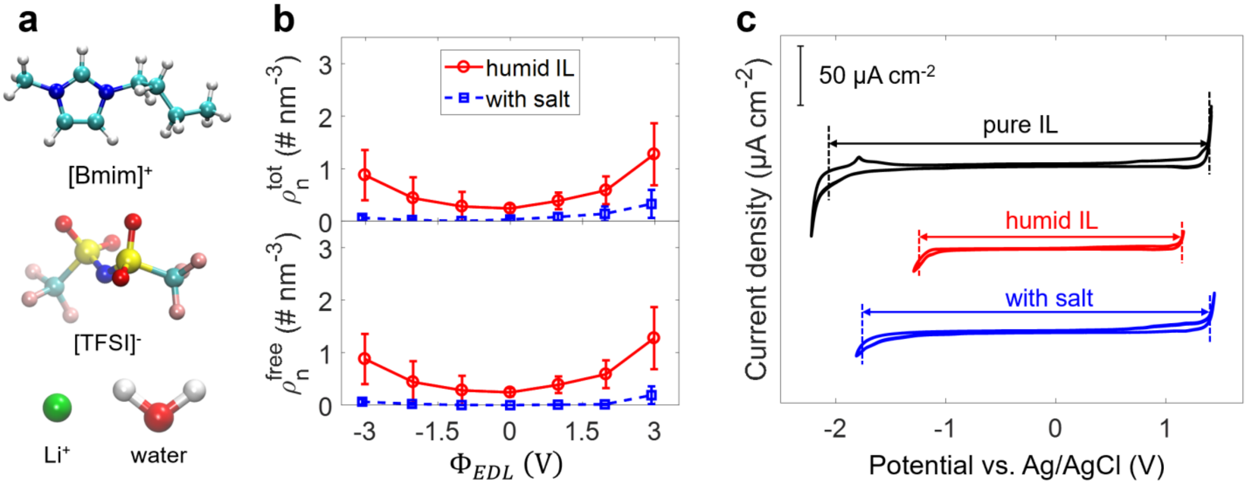

Supplementary Figure 25 | Effect of adding salt on the interfacial water and the electrochemical window. **a**, Molecular structure of cation (1-butyl-3-methylimidazolium, $[Bmim]^+$), anion (bis(trifluoromethylsulfonyl)imide, $[TFSI]^-$), water and lithium ion (Li^+). **b,** Electrosorption of water from humid [Bmim][TFSI] and salt-in-humid [Bmim][TFSI] on electrodes. **c,** Cyclic voltammograms of HOPG in pure [Bmim][TFSI], humid [Bmim][TFSI], and salt-in-humid [Bmim][TFSI] electrolyte. Scan rate: 100 mV s^{-1}. Water contents for humid [Bmim][TFSI] are about 5168 ppm and 5326 ppm for MD simulation and cyclic voltammetry experiments, respectively. The molar salt-water ratio is 1:1.

The origin of the expansion of electrochemical window in salt-in-humid [Bmim][TFSI] has also been investigated by PMF (**Supplementary Figure 26**). PMF curves of Li^+ reveal that Li^+ ions preferentially stay far away from electrode surface. Bound with Li^+, water would be pulled away from the electrode, leading to the decrease of the proportion of interfacial water.

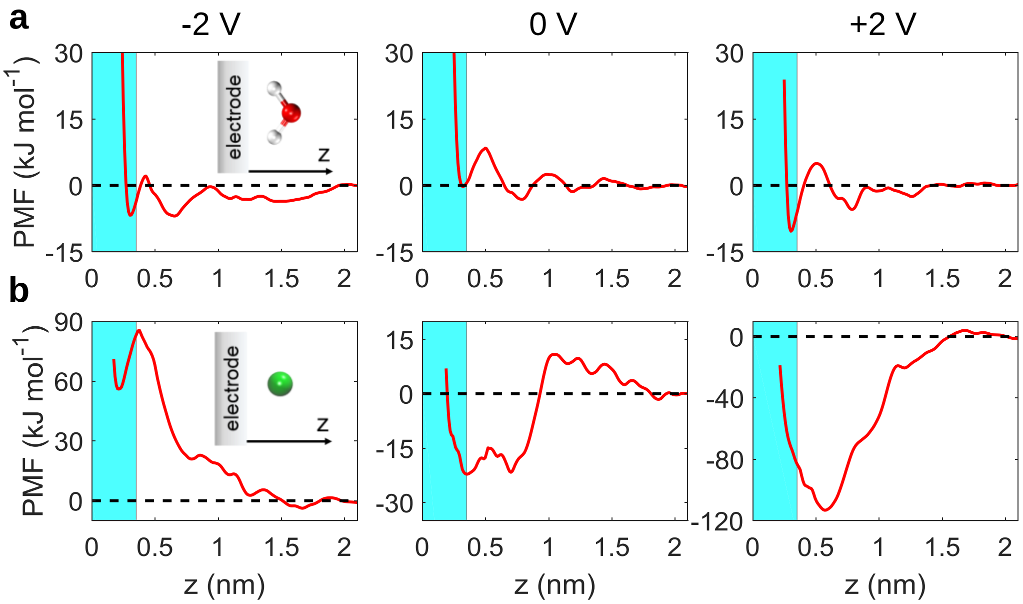

Supplementary Figure 26 | The tendency for water and Li^+ electrosorption at electrode. The potential of mean force (PMF) of water **(a)** and Li^+ **(b)** in 1-butyl-3-methylimidazolium bis(trifluoromethylsulfonyl)imide ([Bmim][TFSI]) as a function of distance from the electrode (z). The PMF curves are calculated via umbrella sampling along the distance from the electrode surface. The cyan shaded region (z < 0.35 nm) is considered to be the interfacial region.

Supplementary References

1 Westrich, H. R. Determination of Water in Volcanic Glasses by Karl-Fischer Titration. *Chemical Geology* **63**, 335-340 (1987).

2 Henderson, W. A. & Passerini, S. Phase Behavior of Ionic Liquid−LiX Mixtures: Pyrrolidinium Cations and TFSI$^-$ Anions. *Chemistry of Materials* **16**, 2881-2885 (2004).

3 Suo, L. *et al.* "Water-in-Salt" Electrolyte Enables High-Voltage Aqueous Lithium-Ion Chemistries. *Science* **350**, 938-943 (2015).

4 McEldrew, M., Goodwin, Z. A. H., Kornyshev, A. A. & Bazant, M. Z. Theory of the Double Layer in Water-in-Salt Electrolytes. *Journal of Physical Chemistry Letters* **9**, 5840-5846 (2018).

5 Dubouis, N. *et al.* The Role of the Hydrogen Evolution Reaction in the Solid–Electrolyte Interphase Formation Mechanism for "Water-in-Salt" Electrolytes. *Energy & Environmental Science* **11**, 3491-3499 (2018).

6 Guillot, B. A Molecular Dynamics Study of the Far Infrared Spectrum of Liquid Water. *The Journal of Chemical Physics* **95**, 1543-1551 (1991).

7 Mart í, J., Padr ó, J. A. & Guardia, E. Computer Simulation of Molecular Motions in Liquids: Infrared Spectra of Water and Heavy Water. *Molecular Simulation* **11**, 321-336 (1993).

8 Bornhauser, P. & Bougeard, D. Intensities of the Vibrational Spectra of Siliceous Zeolites by Molecular Dynamics Calculations. I. Infrared Spectra. *The Journal of Physical Chemistry B* **105**, 36-41 (2001).

9 Praprotnik, M., Janežič, D. & Mavri, J. Temperature Dependence of Water Vibrational Spectrum: A Molecular Dynamics Simulation Study. *The Journal of Physical Chemistry A* **108**, 11056-11062 (2004).

10 Agarwal, V., Huber, G. W., Conner, W. C. & Auerbach, S. M. Simulating Infrared Spectra and Hydrogen Bonding in Cellulose Iβ at Elevated Temperatures. *The Journal of Chemical Physics* **135**, 134506 (2011).

11 Velasco-Velez, J.-J. *et al.* The Structure of Interfacial Water on Gold Electrodes Studied by X-Ray Absorption Spectroscopy. *Science* **346**, 831 (2014).

12 Li, C.-Y. *et al.* In Situ Probing Electrified Interfacial Water Structures at Atomically Flat Surfaces. *Nature Materials* **18**, 697-701 (2019).

13 Suo, L. *et al.* "Water-in-Salt" Electrolyte Makes Aqueous Sodium-Ion Battery Safe, Green, and Long-Lasting. *Advanced Energy Materials* **7**, 1701189-1701198 (2017).

14 Frisch, M. J. et al. *Gaussian 09 Revision D.01* (Gaussian, Inc., Wallingford CT, 2009).

15 Lu, T. & Chen, F. Multiwfn: A Multifunctional Wavefunction Analyzer. *Journal of Computational Chemistry* **33**, 580-592 (2012).

16 Luzar, A. & Chandler, D. Effect of Environment on Hydrogen Bond Dynamics in Liquid Water. *Physical Review Letters* **76**, 928-931 (1996).

17 Vlcek, L. *et al.* Electric Double Layer at Metal Oxide Surfaces: Static Properties of the

Cassiterite−Water Interface. *Langmuir* **23**, 4925-4937 (2007).

18 Kumar, S., Rosenberg, J. M., Bouzida, D., Swendsen, R. H. & Kollman, P. A. The Weighted Histogram Analysis Method for Free-Energy Calculations on Biomolecules. I. The Method. *Journal of Computational Chemistry* **13**, 1011-1021 (1992).

19 Grimme, S., Antony, J., Ehrlich, S. & Krieg, H. A Consistent and Accurate *Ab Initio* Parametrization of Density Functional Dispersion Correction (DFT-D) for the 94 Elements H-Pu. *Journal of Chemical Physics* **132**, 154104 (2010).

20 Kresse, G. & Furthmüller, J. Efficiency of Ab-Initio Total Energy Calculations for Metals and Semiconductors Using a Plane-Wave Basis Set. *Computational Materials Science* **6**, 15-50 (1996).

21 Perdew, J. P., Burke, K. & Ernzerhof, M. Generalized Gradient Approximation Made Simple. *Physical Review Letters* **77**, 3865-3868 (1996).

22 Blöchl, P. E. Projector Augmented-Wave Method. *Physical Review B* **50**, 17953-17979 (1994).

23 Voloshina, E., Usvyat, D., Schutz, M., Dedkov, Y. & Paulus, B. On the Physisorption of Water on Graphene: A CCSD(T) Study. *Physical Chemistry Chemical Physics* **13**, 12041-12047 (2011).

24 Manz, T. A. & Sholl, D. S. Chemically Meaningful Atomic Charges That Reproduce the Electrostatic Potential in Periodic and Nonperiodic Materials. *Journal of Chemical Theory and Computation* **6**, 2455-2468 (2010).

25 Manz, T. A. & Sholl, D. S. Improved Atoms-in-Molecule Charge Partitioning Functional for Simultaneously Reproducing the Electrostatic Potential and Chemical States in Periodic and Nonperiodic Materials. *Journal of Chemical Theory and Computation* **8**, 2844-2867 (2012).

26 Manz, T. A. & Limas, N. G. Introducing DDEC6 Atomic Population Analysis: Part 1. Charge Partitioning Theory and Methodology. *RSC Advances* **6**, 47771-47801 (2016).
